# Asymmetric Brightness Effects With Dark Versus Light Glare-Like Stimuli

**DOI:** 10.1177/2041669521993144

**Published:** 2021-02-25

**Authors:** Yuki Kobayashi, Daniele Zavagno, Kazunori Morikawa

**Affiliations:** School of Human Sciences, 13013 Osaka University, Osaka, Japan; 53347 Japan Society for the Promotion of Science, Japan; Department of Psychology, University of Milano–Bicocca, Milano, Italy; School of Human Sciences, 13013 Osaka University, Osaka, Japan

**Keywords:** lightness/brightness, glare effect, luminance gradient, simultaneous lightness contrast, photometrical reversed glare

## Abstract

The glare effect is a brightness illusion that has captured the attention of the vision community since its discovery. However, its photometrical reversal, which we refer to here as photometrical reversed glare (PRG) stimuli, remained relatively unexplored. We presented three experiments that sought to examine the perceived brightness of a target area surrounded by luminance gradients in PRG stimuli and compare them with conventional glare effect configurations. Experiment 1 measured the brightness of the central target area of PRG stimuli through an adjustment task; the results showed that the target appeared brighter than similar, comparative areas not surrounded by luminance gradients. This finding was unexpected given the recent report that PRG stimuli cause pupil dilation. Meanwhile, Experiments 2 and 3 implemented a rating task to further test the findings in Experiment 1. Again, the study found a robust brightening illusion in the target area of PRG stimuli in a wide range of target and background luminance. The results are discussed in comparison with the brightness enhancement of the glare effect.

Luminance gradients are known to affect lightness and brightness perception (e.g., Cornsweet, 1970; Galmonte et al., 2015; Keil, 2007; Knill & Kersten, 1991; Pereverzeva & Murray, 2014; Soranzo et al., 2009; Zavagno, 2005). One example is the *glare effect* (Zavagno, 1999; Zavagno & Caputo, 2001, 2005), which shows how luminance gradients around a target area influence the target’s perceived brightness (Figure 1A). In this cross configuration, luminance gradients flank a square target area with their light ends touching the target, enhancing its brightness rather strongly. While the glare effect has been the subject of much research (e.g., Correani et al., 2006; Facchin et al., 2017; Hanada, 2012; Kobayashi et al., 2018; Leonards et al., 2005; Lu et al., 2006; Maddaluno et al., 2019), the photometrically reversed version of its cross pattern (hereafter photometrical reversed glare [PRG], Figure 1B), originally introduced by Zavagno (1999) along with the glare effect, has been relatively less studied. Zavagno et al. (2017), who are part of a limited number of studies on PRG stimuli, measured observers’ pupil sizes while they looked at PRG stimuli and found that such patterns caused their pupils to dilate. This finding suggests that PRG stimuli make a target appear darker.

**Figure 1.**
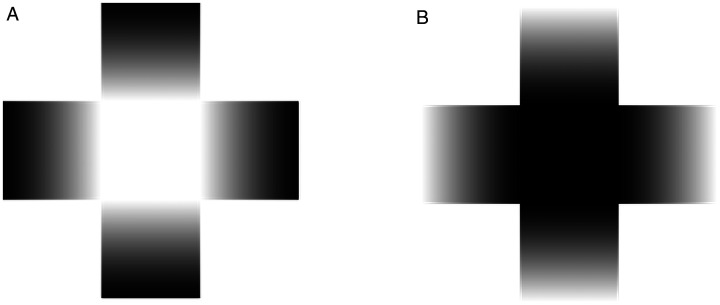
A: A glare stimulus. The white center appears luminous and brighter than the identically white background. B: The photometrical reversed glare (PRG) stimulus.

Agostini and Galmonte (2002) used glare and PRG stimuli to examine whether they affect lightness perception. They introduced small gray targets at the center of the cross configurations (Figure 2), creating a novel pattern for a simultaneous lightness contrast illusion (SLC). They found that the illusion (the apparent difference in lightness between the two equiluminant gray targets in the side-by-side crosses) intensified with respect to classical SLC. This enhancement effect of SLC was recently confirmed by Zavagno et al. (2018).

**Figure 2.**
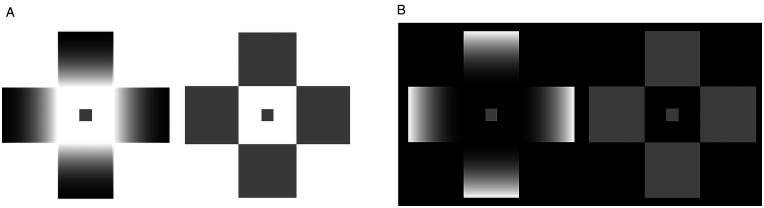
Examples of SLC enhancement. Stronger SLC occurs in gradated crosses. A: The central patch in the left configuration (“increasing gradients”) appears darker than the one on the right. B: The patch on the left (“decreasing gradients”) appears lighter than the one on the right.

SLC enhancement in configurations consisting of glare and PRG stimuli may be explained by the *high-perceptual-contrast hypothesis*. The brighter appearance of a white area surrounded by increasing gradients (gradients with dark outer ends and light inner ends) than an equiluminant area without gradients (Laeng & Endestad, 2012; Tamura et al., 2016) is the basis for the higher perceptual contrast between the central patch and its immediate surrounding in the glare stimulus. One may reasonably believe that such high perceptual contrast affects the lightness of the patch, darkening it when embedded in a glare stimulus and brightening it when embedded in a PRG stimulus. This hypothesis stems from a relatively simple mechanism that does not require complex processing. Gori et al. (2010) found that when the center of a glare-like stimuli appears even brighter because of dynamic presentation (Anstis et al., 2007; Gori & Stubbs, 2006), SLC enhancement also gets stronger (i.e., the central gray patch appears even darker). They claimed that the higher brightness at the center of the glare stimulus causes SLC enhancement, supporting the high-perceptual-contrast hypothesis, which is essentially a percept–percept coupling type of account (Epstein, 1982).

Hence, while studies suggest that the central areas of PRG stimuli appear darker, their brightness has yet to be directly measured. We attempted to fill this gap through three experiments.

In Experiment 1, we examined whether a dark area surrounded by decreasing gradients is perceived as darker (as a convention, we denote the gradients of PRG stimuli as *decreasing* inward from the outer light ends to the inner dark ends while the *increasing* gradients of the glare effect are opposite) and, if so, whether such illusion is accompanied by a perceptible lightness difference in a gray target patch embedded in such configurations. If the dark area appears darkened and the central patch appears lightened simultaneously, such findings would strongly support the high-perceptual-contrast hypothesis, which is consistent with Gori et al. (2010).

Meanwhile, in Experiments 2 and 3, we further examined the brightness of an area surrounded by decreasing gradients (i.e., central area of PRG stimuli) and one surrounded by increasing gradients (i.e., central area of glare stimuli), using several luminance levels for the target area and the background. In these experiments, we implemented a rating task so that the brightness of a target with nearly 0 cd/m^2^ would be measured. We then compared the PRG and glare stimuli in terms of such brightness and its variation due to differences in luminance settings.

## Experiment 1

### Method

#### Participants

Nine naïve volunteers aged 21 to 32 (*M* = 23.9, *SD* = 3.4) participated in this experiment (four female). One female (age 26) did not understand the task and was thus excluded from the analysis. All participants had either normal or corrected-to-normal visual acuity. This study obtained approval from the Research Ethics Committee of the Osaka University School for Human Sciences, and the participants provided written informed consent.

#### Apparatus and Stimuli

The experiment was conducted using a desktop computer (Precision T3500, Dell) and a CRT monitor (Trinitron GDM-F520, Sony) with 1,600 × 1,200 resolution. The luminance levels of the stimuli and background were measured using ColorCAL II (Cambridge Research Systems). Viewing distance was fixed at 57 cm with a chin rest, and the experiment was controlled by PsychoPy2 (Peirce, 2007, 2009; Peirce et al., 2019). The monitor’s gamma was automatically corrected using PsychoPy2’s function based on ColorCAL II’s luminance measurement. The participants performed the main task in a dark room.

**Figure 3.**
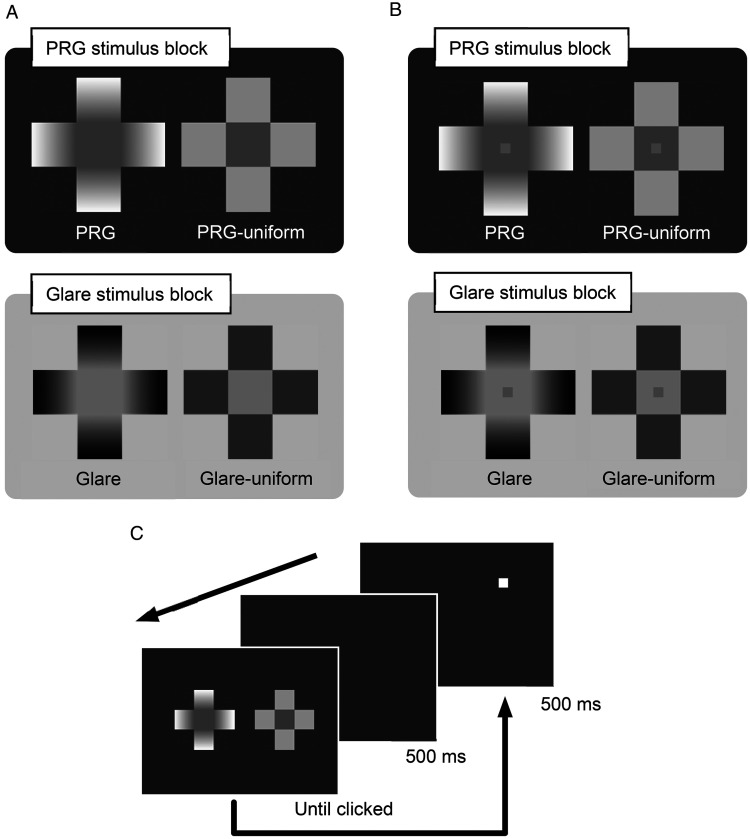
Experiment 1. A: Standard stimuli used in Part 1. B: Standard stimuli used in Part 2. The stimuli in both parts were identical except the lightness patches were present only in Part 2. C: An example trial in Part 1. The marker could appear on the right or the left, indicating the side the comparative (adjustable) stimulus would appear. The trial procedure was the same as in Part 2. PRG = photometrical reversed glare.

The standard stimuli consisted of eight Greek crosses made of five squares (four square arms and a square center; Figure 3A and B); four of the standard stimuli presented a gray square patch (80.4 cd/m^2^, 0.7 cm on a side; Figure 3B) within the central square, and the other four (Figure 3A) were identical to those in Figure 3B except for the presence of the patch. The stimuli measured 9.6 cm in height and width. In the four dark-centered crosses (upper row in Figure 3A and B, hereafter called PRG or PRG-uniform stimuli), the center luminance was fixed at 65.0 cd/m^2^. The decreasing gradients of PRG stimuli ranged linearly from 65.0 cd/m^2^ (inner ends) to 160.8 cd/m^2^ (outer ends). The luminance of the PRG-uniform stimuli’s gray arms was 112.9 cd/m^2^, which was approximately equal to the mean luminance of the PRG stimuli’s gradated arms. In the light-centered crosses (lower row, hereafter called glare or glare-uniform stimuli), the centers were fixed at 96.5 cd/m^2^, and the increasing gradients of glare stimuli ranged linearly from 0.1 cd/m^2^ to 96.5 cd/m^2^. The luminance level of the arms of the glare-uniform stimuli was 48.0 cd/m^2^, which was approximately equal to the mean luminance of those of the glare stimuli. The background was dark (32.2 cd/m^2^) in the PRG stimulus block and light (128.6 cd/m^2^) in the glare stimulus block.

#### Procedure

The entire experiment consisted of two parts. In Part 1, participants viewed stimuli with no central patches; the aim here was to measure brightness in areas surrounded by luminance gradients. In Part 2, participants viewed stimuli with the central gray patch, which sought to evaluate the gradients’ effect on the patch’s lightness. All participants underwent both processes, the order of which was counterbalanced among them.

Part 1 presented PRG and glare stimuli in separate blocks; the order of the two blocks was counterbalanced among the participants. In the PRG stimulus block, a standard stimulus (either a PRG or PRG-uniform stimulus) with a fixed central square luminance was displayed side by side with a comparative stimulus that was identical to the PRG-uniform standard stimulus except that participants could manipulate the central square’s luminance of its central square within a 24.0 to 105.9 cd/m^2^ range. Similarly, in the glare stimulus block, a glare or glare-uniform stimulus was shown with a comparative stimulus that was also identical to the glare-uniform stimulus. The participants could also manipulate the luminance of the central area in the comparative stimulus within a 55.5 to 137.4 cd/m^2^ range. Figure 3C shows an example of one trial. In each trial, one standard and one comparative stimuli were displayed side by side with a 2.4 cm gap between them. The participants’ task was to adjust the luminance of the comparative stimulus’s central area using a mouse wheel to match that of the standard stimulus. When a match was achieved, the participants left-clicked to proceed to the next trial. Between trials, there was a one-second blank during which a square marker appeared (0.5 second) to indicate the side where the adjustable stimulus (comparative stimulus) would appear in the next trial (left or right). This marker was white in the PRG stimulus block and black in the glare stimulus block. In each block, half of the trials followed an ascending series, which initially showed the comparative stimulus at its lowest luminance level, and the remaining half were in a descending series, which initially showed the comparative stimulus at its highest luminance level. Part 1 consisted of 32 trials: 2 blocks × 2 standard stimuli × 2 series (ascending and descending) × 2 presentation positions (left or right) × 2 repetitions. Before the experimental session, the participants practiced with eight trials in a normal-illumination environment.

Part 2 used crosses with central square lightness patches as standard stimuli (Figure 3B) and followed virtually the same procedure as in Part 1. Two blocks were employed: one showing PRG or PRG-uniform stimuli and the other showing glare or glare-uniform stimuli. The standard and comparative stimuli were also displayed side by side. The comparative stimulus was the same as the simultaneously displayed standard stimulus except the luminance of the gray patch in the former could be adjusted within a 48.3 to 112.6 cd/m^2^ range. The participants were to adjust the luminance of the gray patch in the comparative stimulus to match the one in the standard stimulus. The number of trials was the same as in Part 1.

### Results

#### PRG Stimulus Blocks

**Figure 4.**
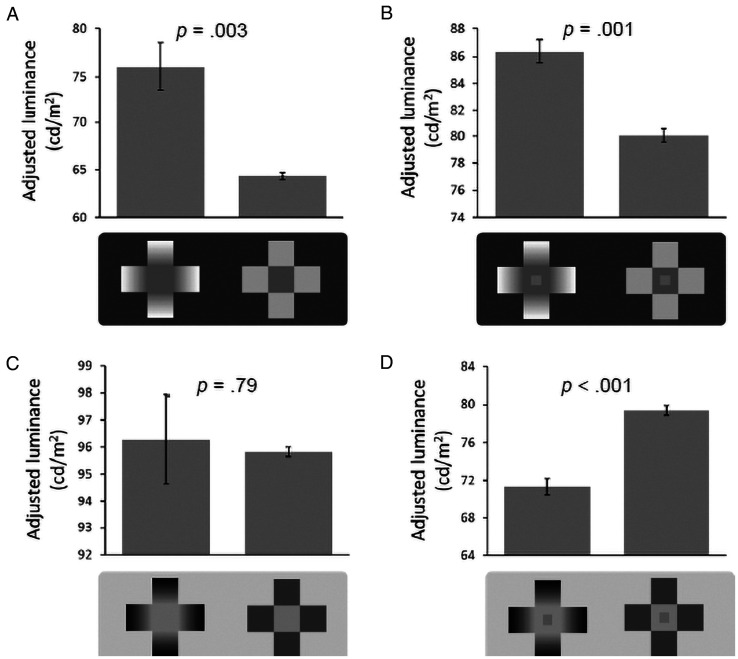
A–D: Results of the adjustments in each block. Error bars indicate *SE*. The patches in the stimuli depicted in B and D are rendered larger than their actual size ratio so that they are more visible in this figure (for the actual size ratio, see Figure 3).

Figure 4 shows the mean results for the PRG and glare stimuli. A few trials (2.3%) were discarded because they were accidentally completed with middle clicks to proceed to a new trial. In all the blocks analyzed here, no significant differences in stimuli position were found (left or right, *p*s > .22), so the data from the left–right positions were pooled in the analyses. Figure 4A shows the results for the PRG stimulus block in Part 1. The mean adjusted luminance levels in the PRG and PRG-uniform stimulus trials were 76.1 cd/m^2^ and 64.3 cd/m^2^, respectively. A two-tailed paired *t*-test showed a significant difference in brightness between the central areas of the PRG and PRG-uniform stimuli—*t*(7) = 4.58, *p* = .003, *d_z_* = 1.62.^1^ A one-sample *t*-test also showed that the adjusted luminance for the PRG stimulus was significantly higher than its physical luminance—65.0 cd/m^2^; *t*(7) = 4.43, *p* = .003, *d_z_* = 1.57. These results showed that in our experiment the dark area surrounded by decreasing gradients was perceived to be brighter, not darker.

Figure 4B shows the results of Part 2. In the PRG stimulus block, the mean adjustments of the PRG and PRG-uniform stimuli patches were 86.4 cd/m^2^ and 80.0 cd/m^2^, respectively. A two-tailed paired *t*-test showed a significant difference between these adjustments, *t*(7) = 5.39, *p* = .001, *d_z_* = 1.91, which is consistent with Agostini and Galmonte’s (2002) results. A one-sample *t*-test on the PRG stimulus also confirmed that it was significantly different from its physical luminance—80.4 cd/m^2^; *t*(7) = 7.00, *p* < .001, *d_z_* = 2.47.

#### Glare Stimulus Blocks

Trials that were accidentally completed using middle clicks were excluded (0.4%). In Part 1, the mean adjusted luminance levels for the glare and glare-uniform stimuli were 96.3 cd/m^2^ and 95.8 cd/m^2^, respectively (Figure 4C). The mean adjustment for the glare stimulus was not significantly different from its physical luminance—96.5 cd/m^2^; *t*(7) = 0.13, *p* = .90, *d_z_* = 0.05—and a paired *t*-test did not show a significant difference between means—*t*(7) = 0.28, *p* = .79, *d_z_* = 0.10. In the present study, we could not confirm the expected brightness enhancement in glare stimuli (Laeng & Endestad, 2012; Tamura et al., 2016) although this might be partly due to the unusually large individual differences as indicated by the very long error bar.

Figure 4D shows the results of Part 2 as well. The mean luminance adjustments for the gray patches in the glare and glare-uniform stimuli were 71.3 cd/m^2^ and 79.4 cd/m^2^, respectively. The adjustment for the glare stimulus was significantly lower than the physical luminance of the patch—80.4 cd/m^2^; *t*(7) = 10.79, *p* < .001, *d_z_* = 3.81. A paired *t*-test also showed a significant difference between means—*t*(7) = 8.98, *p* < .001, *d_z_* = 3.17—which also agrees with Agostini and Galmonte’s results.

### Discussion

According to the results in Experiment 1, the high-perceptual-contrast hypothesis does not explain SLC enhancement. First, the dark area with decreasing gradients appeared brighter than the equiluminant dark area with uniform surroundings (Figure 4A). This brightening effect lowers the perceptual contrast (the apparent difference) between the patch and the central area of the PRG stimulus. Yet SLC enhancement was robustly observed (i.e., the central gray patch in the PRG stimulus appeared lighter in Figure 4B). These results demonstrate that SLC enhancement is not based on the high perceptual contrast between the patch and its surrounding. On the other hand, in the glare stimulus blocks, the results were not conclusive about brightness enhancement in the central area of the glare stimuli because of the large standard error (Figure 4C) although SLC enhancement was clearly present (i.e., the patch appeared darker in Figure 4D).

Whereas previous studies using glare stimuli have suggested that the high-perceptual-contrast hypothesis reasonably accounts for SLC enhancement (Gori et al., 2010; Tamura et al., 2016), the current results from PRG stimuli (Figure 4A and B) were opposite to this hypothesis. To resolve the contradiction, we need an alternative hypothesis because it seems both likely and theoretically parsimonious that the mechanism of SLC enhancement is common between PRG and glare stimuli.

An alternative hypothesis that may explain SLC enhancement is the role of luminance gradients as illumination cues (Zavagno, 2005; Zavagno & Daneyko, 2008) that influence the lightness of the central patches (the albedo hypothesis; see Agostini & Galmonte, 2002) irrespective of their surrounding’s perceived brightness. This suggests that patch lightness is directly affected by luminance gradients and not via the brightness of the central areas of the crosses. In other words, lightness processing may use photometric information (i.e., luminance gradient) independently of the way luminance gradients cause brightness illusions such as the glare effect or brightness enhancement in PRG stimuli.

Our results point to two facts regarding SLC enhancement. First, they demonstrate the robustness of SLC enhancement because we employed an adjustment task instead of the Munsell matching used by Agostini and Galmonte (2002). This indicates that SLC enhancement is sufficiently robust to be observable with different methods. Moreover, the present results showed that SLC enhancement can be observed in a far wider luminance range than the one used by Agostini and Galmonte (our highest luminance was approximately 2.5 times theirs). This is consistent with Zavagno et al. (2018), who found that more illumination and higher luminance strengthens SLC enhancement using paper stimuli. Although illumination that enlarges the visible area may minimize the SLC effect (Agostini & Bruno, 1996), higher luminance seems to intensify this effect as long as it is observed through a CRT monitor in a dark environment. These facts from Experiment 1 may help future studies further investigate SLC enhancement.

For Experiment 1, we generated gradient patterns that were not the standard ones used by the studies we cited; that is, the central areas were either lighter (PRG) or darker (glare) than those usually employed. This was because using the monitor’s lowest or highest luminance would be inappropriate for the adjustment method. For instance, while Zavagno et al. (2017) used a PRG stimulus with a black central area and a black background, the luminance of the PRG stimuli and background in Experiment 1 was fixed at dark gray, which might have affected the illusion’s magnitude and direction. This might explain why we did not obtain the expected brightness effects (Part 1); nevertheless, the presence of gradients affected the perceived lightness of the patch, consistent with other studies (Part 2). Thus, Experiment 2 was conducted to further examine the brightening illusion in the PRG stimuli using various luminance conditions as well as test its robustness.

## Experiment 2

Experiment 2 measured the brightness or darkness of the central areas of the glare and PRG stimuli using various luminance levels for the target areas and background. Moreover, we employed a rating task this time because some of our targets demonstrated a luminance of approximately 0 cd/m^2^, which made it impossible to perform the adjustment.

### Method

#### Participants

Twenty students at the University of Milano–Bicocca participated in Experiment 2 (15 female) whose ages ranged from 20 to 25 years (*M* = 22.3, *SD* = 1.3). All participants had either normal or corrected-to-normal visual acuity and were unaware of the purpose of the experiment. Approval was obtained from the Research Ethics Committee of the University of Milano–Bicocca.

#### Apparatus and Stimuli

The stimuli were displayed with a CRT monitor (LACIE Electron 22 blue II, Mitsubishi Electric Corporation) with a 1,600 × 1,200 resolution and controlled by a laptop computer (Spectre x360, Hewlett-Packard). Although the monitor screen measured 41 cm (width) × 31 cm (height), the experimental program was shown only in a central rectangular area (36 cm × 21.8 cm) on the screen. The remaining periphery remained black (0.1 cd/m^2^) throughout the whole experiment. Luminance levels were measured using a BM-7A (Topcon). The participants viewed the monitor from an approximately 60 cm distance without a chin rest. The experimental program was run on PsychoPy2 (Peirce, 2007, 2009; Peirce et al., 2019), and the monitor’s gamma was corrected using PsychoPy2’s function based on luminance measurement. The participants performed the task in a dark room with black walls where the monitor was the only source of illumination.

#### Stimuli

**Figure 5.**
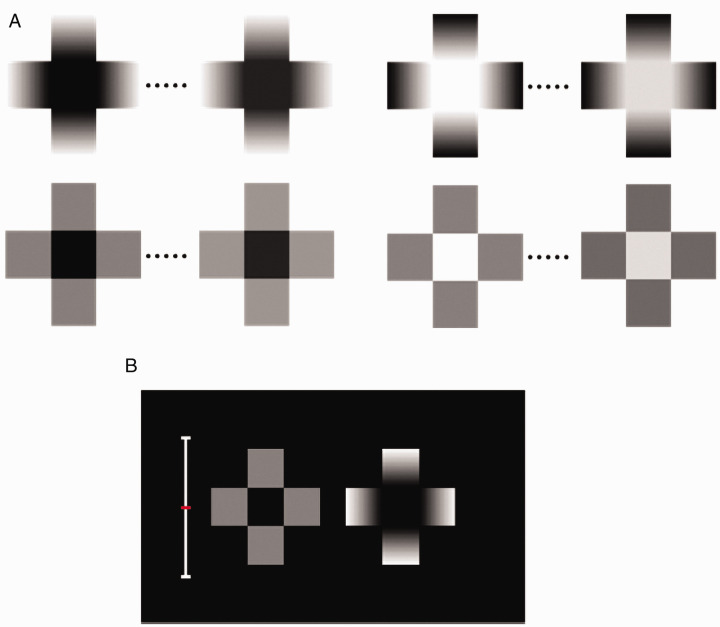
A: Standard (gradated) and comparative (uniform) stimuli used in Experiment 2. Five luminance levels were prepared for each PRG and glare stimulus. B: An example of a trial display.

Experiment 2 used 10 gradated stimuli and 10 uniform stimuli (crosses with solid gray arms; Figure 5A). All had the same shapes and sizes (9 cm width × 9.5 cm height). The 10 gradated stimuli consisted of 5 PRG and 5 glare stimuli. Table 1 shows the luminance levels of their central squares, with the inner ends of their arms having the same luminance as each central square. The outer ends of all PRG stimuli arms were black (0.1 cd/m^2^), and those of all glare stimuli arms were white (43.4 cd/m^2^). The luminance gradient profiles of all gradated stimuli were linear. The 10 uniform stimuli were used as comparative stimuli corresponding to each gradated stimulus. Therefore, each central square of the uniform stimuli had the same luminance levels as those of the corresponding gradated stimuli, and the luminance of the uniform arms was approximately equal to the average luminance of their corresponding gradated arms (see Table 1).

**Table 1.** Luminance of the Central Areas of the Crosses and the Uniform Arms of the Comparative Crosses (cd/m^2^).

		Darkest				Brightest
PRG stimuli	Central areas (inner ends of gradients)	0.1	1.8	3.5	5.2	6.9
	Uniform arms	21.7	22.5	23.4	24.2	25.1
Glare stimuli	Central areas (inner ends of gradients)	36.6	38.3	40.0	41.7	43.4
	Uniform arms	18.3	19.1	20.0	20.8	21.7

*Note.* The luminance level of the central area of the gradated cross was equal to that of its gradients’ inner ends.

#### Procedure

A trial involved displaying a pair of stimuli (a gradated stimulus and a corresponding uniform one) side by side with a 2.1 cm gap (Figure 5B). The uniform stimulus also had a scale beside it. The participants could move the red cursor on the scale using a mouse wheel. Using the cursor and the scale, the participants rated the brightness of the central square of the uniform stimulus compared with that of the gradated stimulus. The participants assessed the uniform stimuli instead of the gradated ones because it was easier for them; the brightness appearance of the gradated stimuli’s center was more difficult to rate directly. The participants were told that the middle of the scale corresponded to the brightness of the central square of the gradated stimulus, and the cursor was initially placed there. The participants were not instructed regarding the criteria of the upper and lower extremities of the scale. Hence, when they perceived the center of a uniform stimulus as darker than that of a gradated one, they should move the cursor downward to indicate how darker it appeared; when it appeared brighter, they should adjust the cursor upward to indicate how brighter the center appeared with respect to the center of the gradated stimulus. Approximately 200 steps were prepared for rating. The participants finished one trial by pressing the space bar. A 1-second blank appeared between trials, which showed nothing but the next trial’s background.

The experiment was divided into two blocks: the PRG stimuli and the glare stimuli. The sequence of the two blocks was counterbalanced among participants. Each block prepared three background luminance levels (dark, mid, and light; PRG stimuli block: 0.1 cd/m^2^, 3.5 cd/m^2^, and 6.9 cd/m^2^, respectively; glare stimuli block: 36.6 cd/m^2^, 40.0 cd/m^2^, and 43.4 cd/m^2^, respectively). The scale was white in the PRG stimuli block and black in the glare stimuli block. Both blocks consisted of 150 trials each: 5 stimuli luminance levels × 3 backgrounds × 2 positions (left or right) × 5 repeats. Before performing the main experiment, all participants performed some practice trials.

### Results

**Figure 6.**
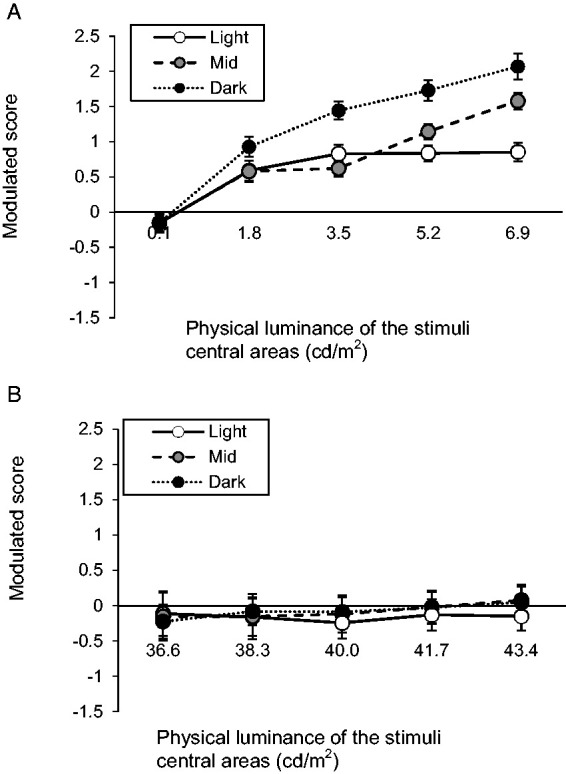
Results of Experiment 2: (A) PRG stimuli block and (B) glare stimuli block. Error bars indicate *SE*.

The two blocks were analyzed separately. The results for the PRG stimuli block are shown in Figure 6A. The rating represents the perceived brightness of the uniform stimuli compared with that of the equiluminant gradated stimuli. In each block, each participant’s rating scores were divided by their scores’ standard deviations. This standardization was necessary because rating scores were based on each participant’s self-defined criteria. To make the results intuitively easier to understand, the negative and positive signs of the ratings were inverted (hereafter referred to as modulated scores); hence, the positive values in Figure 6A and B mean that the gradated stimuli were perceived as brighter than the corresponding uniform ones. The central area of the PRG stimuli generally appeared brighter than the equiluminant area surrounded by uniform gray, except for those with the darkest physical luminance. One-sample *t*-tests^2^ were performed for each condition of the stimuli’s luminance to test the brightening illusion caused by decreasing gradients, which supported the aforementioned observation—0.1 cd/m^2^: *t*(19) = 1.21, *p* = 0.24, *d_z_* = 0.27; 1.8 cd/m^2^: *t*(19) = 5.05, *p* < .001, *d_z_* = 1.13; 3.5 cd/m^2^: *t*(19) = 9.00, *p* < .001, *d_z_* = 2.01; 5.2 cd/m^2^: *t*(19) = 11.47, *p* < .001, *d_z_* = 2.56; 6.9 cd/m^2^: *t*(19) = 12.12, *p* < .001, *d_z_* = 2.71. Moreover, the data underwent a two-way repeated-measures ANOVA^3^ (Background Luminance × Stimuli’s Physical Luminance), which showed that both factors had a significant effect on brightness ratings—background luminance: *F*(1. 23, 23.3) = 70.73, *p* < .001, η_p_^2^ = 0.79; stimuli luminance: *F*(2.61, 49.5) = 67.76, *p* < .001, η_p_^2^ = 0.78—and a significant interaction—*F*(3.71, 70.5) = 16.88, *p* < .001, η_p_^2^ = 0.47. The magnitudes of brightening illusion in the PRG stimuli center varied depending on background luminance; that is, they were greater in darker backgrounds—dark and mid: *t*(19) = 8.30, *p* < .001, *d_z_* = 1.85; mid and light: *t*(19) = 5.63, *p* < .001, *d_z_* = 1.26. As for the main effect of the stimuli luminance, the illusion magnitudes were enhanced in brighter stimuli—0.1 cd/m^2^ and 1.8 cd/m^2^: *t*(19) = 6.25, *p* < .001, *d_z_* = 1.40; 1.8 cd/m^2^ and 3.5 cd/m^2^: *t*(19) = 4.01, *p* = .002, *d_z_* = 0.90; 3.5 cd/m^2^ and 5.2 cd/m^2^: *t*(19) = 4.49, *p* < .001, *d_z_* = 1.00; 5.2 cd/m^2^ and 6.9 cd/m^2^: *t*(19) = 3.92, *p* = .002, *d_z_* = 0.88. The simple main effects of the background luminance were significant in the 1.8 to 6.9 cd/m^2^ conditions (*p*s < .001) but not in the 0.1 cd/m^2^ condition (*p* = .996). In the 3.5 cd/m^2^ condition, the general trend was reversed; the mid-background condition appeared darker than the light-background condition—*t*(19) = 2.42, *p* = .026, *d_z_* = 0.54—probably because the target area had the same luminance as the background and was integrated into it.

Figure 6B shows the results for the glare stimuli block, whose general trend appears completely different from that of the PRG stimuli block. One-sample *t*-tests showed no significant difference from zero in any stimuli physical luminance condition—*t*s(19) < 0.69, uncorrected *p*s > .50, *d*_z_s < 0.15; that is, it is as if participants did not experience a brightness illusion. Moreover, a two-way repeated-measures ANOVA also showed no significant effect—main effect of background luminance: *F*(1.11, 21.2) = 0.35, *p* = .59, η_p_^2^ = 0.02; main effect of stimuli luminance: *F*(1.32, 25.1) = 0.66, *p* = .47, η_p_^2^ = 0.03; interaction between both factors: *F*(6.9, 131.1) = 1.05, *p* = .40, η_p_^2^ = 0.05. Background and stimuli luminance did not affect the difference in brightness between gradated and uniform stimuli.

### Discussion

Experiment 2 showed that the central area of the PRG stimuli generally appears brighter than an equiluminant area with uniform gray arms, confirming the results of Experiment 1. The illusion direction reversed only in the darkest stimuli, whose central target area was black (0.1 cd/m^2^), with the target areas perceived slightly darker than the comparative stimuli although the difference was not significant.

Regardless of stimuli and background luminance, glare stimuli were not perceived significantly brighter than equiluminant areas surrounded by uniform gray arms. This result was also consistent with those in Experiment 1. Different from the present outcomes, one study found a brightening illusion of glare stimuli across a wide luminance range (Tamura et al., 2016). The difference between those results and ours call for further investigation.

Experiments 1 and 2 suggested a visual effect asymmetry caused by decreasing and increasing gradients. While glare stimuli may appear brighter or not cause a brightness illusion, PRG stimuli also appear brighter, not darker. In Experiment 2, despite the symmetrically reversed luminance settings in the two experimental blocks, the results showed an asymmetric tendency.

Experiment 2 also showed an asymmetry between PRG and glare stimuli in their susceptibility to background and stimuli luminance (i.e., the main effects of the background and stimuli luminance were significant in the PRG stimuli block while neither were significant in the glare stimuli block). However, as widely known, human eyes are less sensitive to fixed brightness increments at high luminance than at low luminance (i.e., *Weber’s law*); therefore, the strength of luminance effects on the illusion magnitude of the PRG stimuli and the weakness of its effects on that of the glare stimuli might be attributed to sensory level (i.e., in terms of sensory physiology, the differences in the background and stimuli luminance levels were smaller for the glare stimuli block than for the PRG stimuli block in Experiment 2). Therefore, to clarify the asymmetry between the effects of increasing and decreasing gradients, Experiment 3 was conducted.

## Experiment 3

Experiment 3 was similar to Experiment 2 except for the different luminance levels of the background and target areas. We used precisely the same target–background luminance combinations for both the PRG and glare stimuli to eliminate the possible effect of the Weber ratio. Moreover, the background luminance range was much larger in this experiment than in Experiment 2 to further clarify its influence.

### Method

#### Participants

Twenty Osaka University students participated in Experiment 3 (nine female). Their ages ranged from 20 to 31 years (*M* = 22.9, *SD* = 2.6). They all had either normal or corrected-to-normal visual acuity and were unaware of the experiment’s purpose. Approval was provided by the Research Ethics Committee of the Osaka University School for Human Sciences, and the participants provided written informed consent.

#### Apparatus, Stimuli, and Procedure

**Figure 7.**
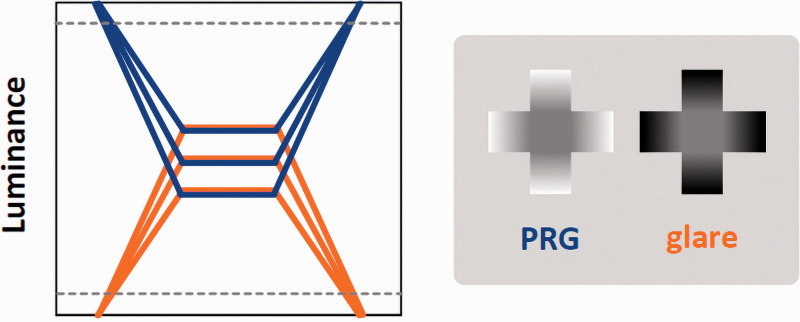
Schematic illustration of the stimuli luminance profiles in Experiment 3. Blue lines indicate the three PRG stimuli profiles, and orange lines indicate the glare stimuli profiles. They were symmetric, and the PRG and glare stimuli had the same central area luminance. Gray dashed lines indicate background luminance. Both background luminance conditions were used for both the PRG and glare stimuli blocks. PRG = photometrical reversed glare.

This experiment used the same apparatus in Experiment 1 and followed the same experimental procedure in Experiment 2. The stimuli were almost the same as those in Experiment 2 but with different luminance levels. The luminance of the central areas of the PRG and glare stimuli was the same: 37.9 cd/m^2^, 47.4 cd/m^2^, and 56.8 cd/m^2^. The highest luminance in all three PRG stimuli (outer ends of the decreasing gradient) was 94.6 cd/m^2^ while the lowest luminance in all three glare stimuli (outer ends of the increasing gradient) was 0.1 cd/m^2^. Figure 7 schematically illustrates stimuli luminance. Stimuli size was slightly different from that in Experiment 2 (9.6 cm in height and width in Experiment 3). Two luminance levels (6.4 cd/m^2^ and 88.3 cd/m^2^) were used as backgrounds in the PRG and glare stimuli blocks, each of which consisted of 48 trials: 3 stimuli luminance levels × 2 backgrounds × 2 positions × 4 repeats.

### Results

**Figure 8.**
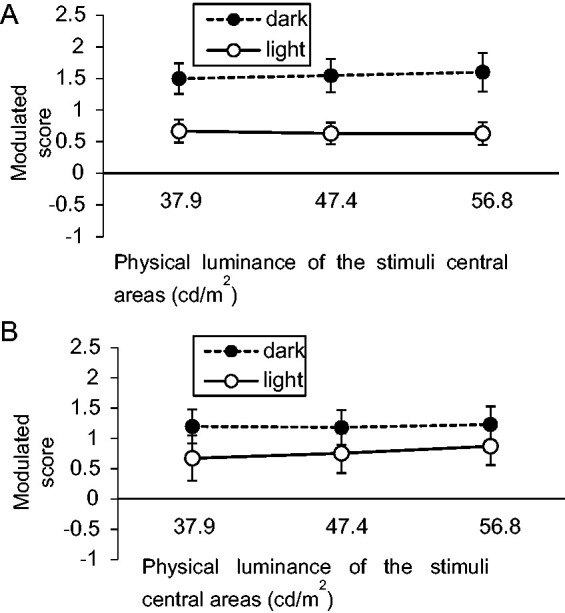
Results of Experiment 3: (A) PRG stimuli block and (B) glare stimuli block. Error bars indicate *SE*.

The results (Figure 8) were analyzed the same way as in Experiment 2; that is, positive values mean that the gradated stimuli (PRG or glare) were perceived brighter. Figure 8A shows the results for the PRG stimuli block, and Figure 8B displays those for the glare stimuli block. In both blocks, the center of the gradated stimuli appeared brighter than that of the uniform stimuli. One-sample *t*-tests were performed with background conditions pooled and confirmed that the ratings for the three luminance conditions were significantly above zero in both blocks—PRG dark: *t*(19) = 5.62, *p* < .001, *d_z_* = 1.26; PRG mid: *t*(19) = 5.24, *p* < .001, *d_z_* = 1.17; PRG light: *t*(19) = 4.77, *p* < .001, *d_z_* = 1.07; glare dark: *t*(19) = 2.94, *p* = .008, *d_z_* = 0.66; glare mid: *t*(19) = 3.28, *p* = .008, *d_z_* = 0.73; glare light: *t*(19) = 3.55, *p* = .006, *d_z_* = 0.79; correction via Holm’s (1979) method was separately performed on each block. Two-way repeated-measures ANOVAs were performed on the two blocks, resulting in significant main effects of background for both blocks—PRG: *F*(1, 19) = 25.52, *p* < .001, η_p_^2^ = .57; glare: *F*(1, 19) = 4.76, *p* = .042, η_p_^2^ = .20—but nonsignificant main effects of stimuli luminance and interactions also for both blocks—PRG stimuli luminance: *F*(1.88, 35.8) = 0.06, *p* = .93, η_p_^2^ = .003; PRG interaction: *F*(2, 38) = 0.40, *p* = .67, η_p_^2^ = .02; glare stimuli luminance: *F*(1.34, 25.5) = 0.58, *p* = .50, η_p_^2^ = .03; glare interaction: *F*(1.92, 36.5) = 0.56, *p* = .57, η_p_^2^ = .03.

### Discussion

Experiment 3 adopted the same background–stimuli luminance combinations for both the PRG and glare stimuli to disregard the influence of the Weber ratio, and the two blocks displayed virtually the same tendencies. In Experiment 2, the effects of background and stimuli luminance on illusion magnitudes showed different trends in the PRG and glare stimuli blocks, but Experiment 3 indicated that this difference was largely due to different sensitivities to high and low luminance. More importantly, however, Experiment 3 clearly demonstrated asymmetry between the two blocks, which Experiments 1 and 2 suggested: regardless of photometric reversal, both decreasing and increasing gradients caused a robust brightening illusion in all luminance conditions. If the effects were symmetrical, one would expect a darkening effect with PRG stimuli.

Brightness enhancement caused by increasing gradients (Laeng & Endestad, 2012; Tamura et al., 2016) was not clear in Experiment 1 and 2 but observable in Experiment 3. This difference may stem from the integration of target areas into the background. In Experiment 3, the luminance difference between the target area and background was large, and thus, the target areas clearly contrasted the background while the lesser luminance contrast in Experiments 1 and 2 made the target areas appear to almost merge with the background. Tamura et al. (2016) and Laeng and Endestad (2012) used circular glare stimuli, which clearly distinguished the central area from its background. The experiments in this study suggest that the distinction between the center and background of the glare stimuli is an important factor for the brighter appearance of the glare. In fact, the glowing appearance of glare stimuli can also be achieved with so-called subjective contours, as with the Kanizsa triangle (Zavagno, 1999; Zavagno & Daneyko, 2017) where physically absent but perceptually present contours appear to glow, thus separating the glowing triangle from the equiluminant background.

## General Discussion

The present study focused on how the brightness of the central area of PRG stimuli is perceived. Experiment 1, which measured perceived brightness in gradated stimuli centers and the SLC illusion, found that the central area of the PRG stimuli appears brighter than an equiluminant area with uniform arms and that high perceptual contrast does not cause SLC enhancement. Although studies (Gori et al., 2010; Tamura et al., 2016) suggested the high-perceptual-contrast hypothesis accounts for SLC enhancement, in the present study SLC enhancement was observed even with reduced perceptual contrast because of the enhanced brightness of the PRG stimuli center. Experiment 1 suggested asymmetric illusory effects from PRG and glare stimuli, and Experiment 2 examined these effects using several luminance conditions for stimuli and background. Again, the central areas of the PRG stimuli appeared generally brighter than equiluminant areas with uniform arms whereas the same areas of the glare stimuli did not yield a brightening illusion. In Experiment 2, stimuli and background luminance strongly influenced the illusion in the PRG stimuli but not in the glare stimuli. Experiment 3, meanwhile, indicated that the asymmetry in the effect of stimuli and background luminance was largely due to the Weber ratio but also demonstrated the asymmetric illusory effect in the PRG and glare stimuli more clearly than Experiment 2. Both PRG and glare stimuli enhanced brightness despite their completely reversed luminance configurations. These experiments showed robust brightness enhancement due to decreasing gradients, which suggests that (a) the high-perceptual-contrast hypothesis does not explain SLC enhancement, and (b) illusory effects resulting from decreasing/increasing gradients are asymmetric in that they both enhance brightness while decreasing gradients were expected to cause brightness darkening.

Although the present study offered new findings on the effects of gradients, the mechanisms remain largely unexplained. First, the brightening illusion mechanism in the PRG stimuli is obscure. The albedo hypothesis (e.g., Agostini & Galmonte, 2002; Soranzo & Agostini, 2004) may explain our findings: An area surrounded by decreasing gradients is likely to be interpreted as shaded, and the visual system may well estimate its reflectance as higher than it appears by discounting the influence of reduced illumination. If such a hypothesis were true, the brighter appearance of PRG stimuli should be considered a lightness illusion of surface reflectance and not a brightness illusion of perceived illumination. However, a subjective impression of PRG stimuli is more like a foggy cave than a light-reflecting surface, and the albedo hypothesis cannot solely explain the effect of background luminance on the magnitude of such an illusion. This newly found asymmetry between illusions would provide important suggestions regarding the role of luminance gradients in brightness/lightness perception but would also require further investigation.

From the results of Experiments 1 and 2, one may be tempted to conclude that glare stimuli do not robustly appear luminous, but such a statement would be premature. In this study, we measured the darkness/brightness appearance of gradated stimuli by comparing it with that of nongradated stimuli. This method emphasized the glare effect’s quantitative aspect rather than its qualitative visual effect, that is, its glowing appearance (i.e., luminosity). In fact, Zavagno and Caputo (2005) and Kobayashi et al. (2018) have shown that luminosity is perceived at much lower luminance than the appearance of white when the center is surrounded by increasing gradients. Our participants may well have perceived luminosity in the glare stimuli even though they appeared no brighter than the nongradated stimuli. Nevertheless, as stated earlier, the brightening effect found for the PRG stimuli remains rather unknown and unexpected.

Our experiment results demonstrate the importance of luminance gradients as information used by the visual system to process brightness and lightness. While much research has addressed how the visual system deals with gradients, several unexplored aspects remain. The present study investigated how decreasing gradients affect a target area and discovered an unexpected brightening effect. Future research should focus on the asymmetry between increasing and decreasing gradients to better understand the mechanisms involved in both brightness and lightness perception.
